# Impact of Neuroprotection on Incidence of Alzheimer's Disease

**DOI:** 10.1371/journal.pone.0000052

**Published:** 2006-12-20

**Authors:** Raúl de la Fuente-Fernández

**Affiliations:** Section of Neurology, Hospital A. Marcide Ferrol, Spain; Laboratory of Neurogenetics, National Institutes of Health, United States of America

## Abstract

Converging evidence suggests that high levels of education and intellectual activity increase the cognitive reserve and reduce the risk of dementia. However, little is known about the impact that different neuroprotective strategies may have on the incidence of Alzheimer's disease. Using a simple mathematical regression model, it is shown here that age-specific counts of basic cognitive units (surrogate of neurons or synapses) in the normal population can be estimated from Alzheimer's incidence rates. Hence, the model can be used to test the effect of neuroprotection on Alzheimer's incidence. It was found that the number of basic cognitive units decreases with age, but levels off in older people. There were no gender differences after correcting for survival. The model shows that even modest neuroprotective effects on basic cognitive units can lead to dramatic reductions in the number of Alzheimer's cases. Most remarkably, a 5% increase in the cognitive reserve would prevent one third of Alzheimer's cases. These results suggest that public health policies aimed at increasing the cognitive reserve in the general population (e.g., implementing higher levels of education) are likely the most effective strategy for preventing Alzheimer's disease.

## Introduction

There is concern that the progressive loss of neurons and synapses with age may lead to dramatic increases in incidence rates of late-onset Alzheimer's disease in future generations with greater longevity [1]. However, while there is substantial evidence that normal aging is associated with some cognitive impairment, the relationship between Alzheimer's disease, normal aging, and brain function is still far from clear [2]–[5]. It has been shown that most people accumulate Alzheimer-related lesions with age [6], but there is still controversy as to whether neuron death (or synapse density loss) is an inevitable result of normal aging [7]. Conversely, advanced Alzheimer's pathology is sometimes associated with normal cognition [8]. These observations highlight a considerable disjunction between pathology and function in the aging brain, and support the notion that the cognitive reserve plays a crucial role in modifying the relationship between pathology and cognitive function [9], [10]. Indeed, there is converging evidence that education and cognitive activity have protective effects on cognition, reducing the risk of dementia [11]–[14].

The main objective of this study was to evaluate the effect of neuroprotection on Alzheimer's disease incidence. From a functional viewpoint, normal cognition depends on the contribution of a number of basic cognitive units (BCU), which may represent neurons, synapses or cerebral circuits (biological units). The cognitive reserve refers to the number of BCU an individual has to lose before developing dementia symptoms [10]. Age-specific incidence rates of Alzheimer's disease reflect the instantaneous probability of BCU counts dropping below a certain threshold, which separates normal cognition from dementia. Consequently, reported incidence rates of Alzheimer's disease can be used to model and ‘visualize’ the effect of aging on BCU counts in the normal (living) population. Most importantly, this simple mathematical model offers a unique method to estimate the effect of neuroprotection on Alzheimer's disease incidence. Several observations support the model: 1) Virtually the entire population has Alzheimer-related pathology (amyloid plaques and neurofibrillary tangles) by age 90 years [6]; 2) The hippocampus is targeted by both normal aging and Alzheimer's disease [7]; and 3) Synapse density loss seems to be the major correlate of cognitive impairment in both normal aging and Alzheimer's disease [11].

## Methods

### Data: Epidemiological studies

A Medline/PubMed search of the English literature between 1966 and the end of 2005 identified 26 studies on the incidence of Alzheimer's disease that met the inclusion criteria. Studies were selected according to the following inclusion criteria: 1) Case finding was based on a field survey with population-based samples; 2) Males and females were included in the study; 3) Age-specific incidence rates of Alzheimer's disease were reported for dementia from mild to severe; 4) Standard errors of incidence rates were reported or could be calculated from the reported data. There were 11 studies from Europe [15]–[25], 8 studies from North America [26]–[33], 4 studies from Asia [34]–[37], 1 study from Africa [38], 1 study from Australia [39], and 1 study from South America [40]. Sixteen studies included specific data for women and men. Some of these 26 studies were included in previous meta-analyses and review articles [41]–[45].

### Mathematical model

The model assumes that 1) BCU counts are approximately normally distributed at any given age, and 2) Alzheimer's disease occurs when BCU counts drop below a certain threshold value (symptom threshold). Therefore, as incidence rates of Alzheimer's disease represent the probability (*p*) of failure in cognition at any given age, age-specific *z*-scores can be directly obtained from these *p* values. Re-arranging the formula of the *z*-score, the following equation is obtained: E(*y*|*x*) = *c*−*z*-score×SD(*y*|*x*), where *c* represents the symptom threshold, E(*y*|*x*) is the expected BCU count (*y*) at any given age (*x*), and SD(*y*|*x*) is the corresponding standard deviation.

It should be noted that post-mortem studies support the model assumption that BCU counts are approximately normally distributed at any given age, and suggest that Alzheimer's symptoms appear when the BCU count drops to about 50% the normal value at an early age (say, age 20 years) [11], [46]–[51]. Hence, the BCU count can be expressed as a proportion, using *c* = 0.5 as the most likely threshold value. Regression analyses of previous post-mortem studies also suggest that SD(*y*|*x*) = 0.1 is the most likely value for the standard deviation. Consequently, the final equation is E(*y*|*x*) = BCU|age = 0.5−0.1×*z*-score. Sensitivity analyses were carried out to evaluate the effect of changing these parameter values.

### Statistical analysis

Average annual age-specific incidence rates of Alzheimer's disease (per 1,000 person-years) were estimated from the reported incidence rates using two different non-parametric regression methods, *loess* and *sm* [52]–[54]. As rates are always restricted (i.e., they can only take positive values), a log transformation was used in the regression analysis, where log is the natural logarithm. The estimated age-specific average values were then converted back to the original scale, which gives the average *p* at any given age, from which the corresponding *z*-scores were directly obtained. These *z*-scores were then incorporated into the final equation BCU|age = 0.5−0.1×*z*-score, obtaining thus the BCU curve. The smoothing parameter was selected according to the cross-validation criterion in each regression analysis [52]–[54]. The inverse variances of log incidence rates of Alzheimer's disease were used as weights, which are proportional to the number of incident Alzheimer's cases [55]. The same non-parametric weighted regression methods were also applied to the different E(*y*|*x*) values obtained directly from the reported incidence rates for each study. Gender comparisons were performed using weighted non-parametric methods (*sm* ancova and generalized additive models using *loess*) [53].

## Results

### Estimates of basic cognitive units (BCU)

**Figure 1:**
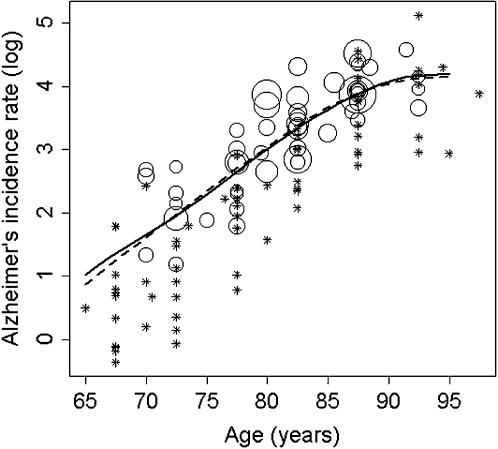
Non-parametric regression of the natural logarithm (log) of Alzheimer's incidence rates on age. Rates were estimated per 1,000 person-years. The diameter of the circles for each data point is proportional to the square root of the number of Alzheimer's cases. For visibility, very small circles have been replaced by asterisks. The solid and dashed curves were obtained by weighted non-parametric regression methods using *sm* and *loess*, respectively (see Methods); the smoothing parameter corresponded to span = 1 in both cases [52]–[54]. The two curves closely agree and depart significantly from a linear model (*sm* regression, *P*<0.0001). There was no evidence for non-constant variance (score test, *P* = 0.88).

**Figure 2:**
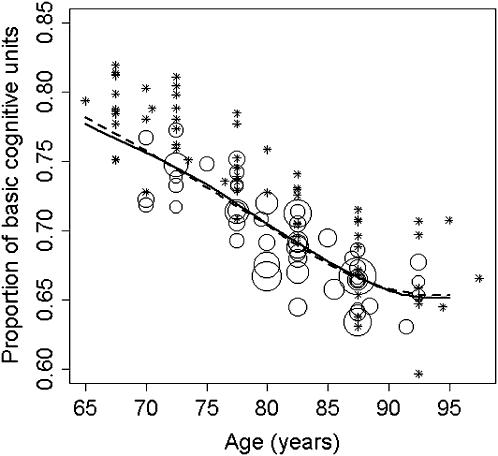
Non-parametric regression of the number of basic cognitive units (BCU) on age. The BCU count is expressed as a proportion with respect to the average normal BCU count at an early age (say, age 20 years). Again, circle sizes are proportional to the square root of the number of Alzheimer's cases, asterisks represent very small circles, the solid curve was obtained using *sm*, and the dashed curve was obtained using *loess* (span = 1). Data points were directly derived from the *z*-scores associated with the corresponding Alzheimer's incidence rates, using the equation: BCU|age = 0.5−0.1× *z*-score, where the threshold (*c*) is 0.5, and the standard deviation (SD) is 0.1 (see Methods). The plotted curves were derived from the average annual incidence rates of Alzheimer's disease (see Fig. 1), but virtually identical curves were obtained by direct weighted non-parametric regression of BCU points on age. Again, both BCU curves closely agree and depart significantly from linearity (*sm* regression, *P*<0.0001). Thus, BCU counts decrease with age up to approximately age 85 years, and then level off.

Non-parametric fits of log incidence rates of Alzheimer's disease on age (Fig. 1), and the corresponding BCU curves (Fig. 2), were found to depart significantly from linearity (*sm* regression, *P*<0.0001). Thus, BCU counts decrease with age in the normal population up to around age 85 years and then tend to level off. Sensitivity analysis demonstrated consistency in the overall pattern of the BCU curve i) after changing the values for *c* and SD, ii) using age-dependent SD values (either age-related increase or age-related decrease of SD), and iii) using non-constant *sm* smoothing parameters.

**Figure 3:**
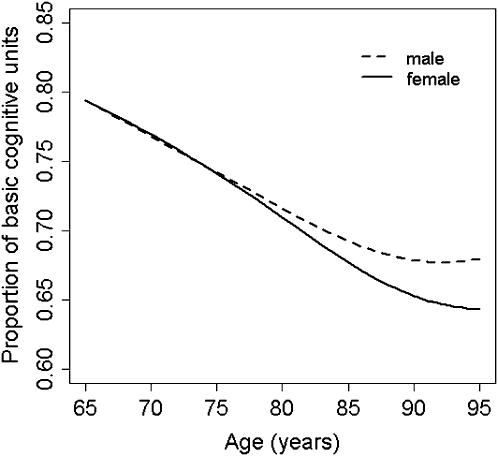
Non-parametric regression curves of basic cognitive units (BCU) on age, for women and men. The overall pattern of these two BCU *loess* curves was similar to that found for the total population (see Fig. 2), with a leveling off at older ages. However, there were statistically highly significant differences between women and men (generalized additive model for log incidence rates of Alzheimer's disease, *P* = 0.0015). While the two curves are very similar up to age 75 years, women deteriorate more than men at older ages.

**Figure 4:**
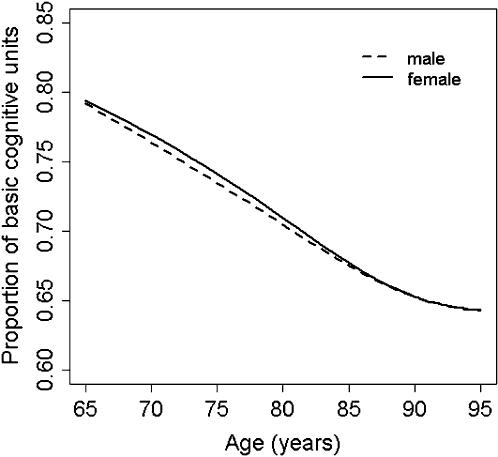
Adjusted non-parametric regression curves of basic cognitive units (BCU) on age, for women and men. There were no statistically significant differences in BCU curves between women and men after correcting for gender differences in survival (generalized additive model for log incidence rates of Alzheimer's disease, *P* = 0.16).

The BCU curves obtained for women and men also showed a similar overall pattern, with a leveling off at advanced age (Fig. 3). However, while the female and male BCU curves run in close proximity up to age 75 years, women deteriorate further at older ages. This difference was statistically significant (for log incidence rates of Alzheimer's disease: *sm* ancova, *P*<0.001; generalized additive model using *loess, P* = 0.0015). Remarkably, the two BCU curves become virtually identical at older ages (Fig. 4) after crude adjustment for gender differences in survival using general population data (generalized additive model for log incidence rates of Alzheimer's disease including age-specific female/male survival ratios as a covariate, *P* = 0.16) [56], [57].

### Effect of neuroprotection

Two different types of neuroprotection were tested: 1) Cell-rescuing therapies (Protect-1), which lead to increased number of BCU at any given age; and 2) Neuroprotective strategies aimed at increasing the cognitive reserve (Protect-2). The model shows that for 5% neuroprotection in age-specific BCU counts (Protect-1), the total number of Alzheimer's cases is expected to decrease by 45% between ages 65 and 94 years (Table 1); for 20% neuroprotection, 96% of Alzheimer's cases would be prevented. On the other hand, a 5% increase in the cognitive reserve (i.e., lowering of the threshold value from 0.5 to 0.475; Protect-2) would save 34% of Alzheimer's cases in the age range 65–94 years (Table 1).

**Table 1:**
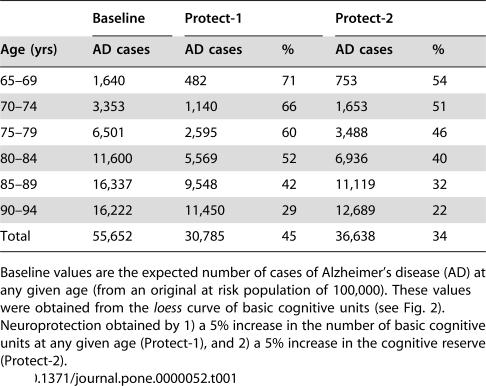
Effect of neuroprotection on Alzheimer's disease (AD)

	Baseline	Protect-1		Protect-2	
Age (yrs)	AD cases	AD cases	%	AD cases	%
65–69	1,640	482	71	753	54
70–74	3,353	1,140	66	1,653	51
75–79	6,501	2,595	60	3,488	46
80–84	11,600	5,569	52	6,936	40
85–89	16,337	9,548	42	11,119	32
90–94	16,222	11,450	29	12,689	22
Total	55,652	30,785	45	36,638	34

Baseline values are the expected number of cases of Alzheimer's disease (AD) at any given age (from an original at risk population of 100,000). These values were obtained from the *loess* curve of basic cognitive units (see Fig. 2). Neuroprotection obtained by 1) a 5% increase in the number of basic cognitive units at any given age (Protect-1), and 2) a 5% increase in the cognitive reserve (Protect-2).

## Discussion

This study shows that the incidence rate of Alzheimer's disease increases with age but slows down at older ages. Although not all [42], most previous meta-analyses found a similar deceleration in incidence rates of Alzheimer's disease [41], [43], [44]. The model consistently shows that the number of BCU decreases with age but tends to reach a plateau at advanced age. This observation likely reflects the combination of two different phenomena: 1) The earlier development of dementia in subjects at higher risk (e.g., carriers of the apolipoprotein ε4 allele), which then gives rise to a healthier normal population; and 2) A within-subject constant (or decreasing) risk of neuron death with age, which has experimentally been shown to govern cell kinetics in Alzheimer's disease and other neurodegenerative disorders [58]. It should be emphasized that while different threshold and variance values give different BCU curves, the overall pattern of the curve (i.e., a leveling off at older ages) is mostly independent of these parameter values and consistently suggests a decreasing risk of neuron death at advanced age. In fact, Fig. 3 shows that the average BCU count may indeed increase in older men. This result is in keeping with recent post-mortem results [59], and suggests that men surviving to advanced age might represent a ‘super-normal’ subpopulation [60].

The model also shows gender differences in BCU curves. There is experimental evidence suggesting that estrogens likely have a protective effect on cognitive function [7]. Therefore, it could be argued that estrogen deprivation at advanced age might explain why women have in general higher incidence rates of Alzheimer's disease compared to men [41]–[45]. However, the overall pattern of the BCU curves (i.e., no gender difference up to age 75 years) is difficult to reconcile with this notion. Likewise, constant gender differences in the cognitive reserve (e.g., differences in the level of education) should be associated with differences in BCU curves at any given age, not simply in the older range. The most likely explanation is a survival effect. Indeed, the female and male BCU curves become virtually identical at older ages after correcting for gender differences in survival (Fig. 4). This observation suggests that, in relative terms, socio-economic growth and improvements in public heath care policies and services may tend to favor the survival of relatively frail subjects, particularly at advanced age. Hence, incidence rates of late-onset Alzheimer's disease are likely to increase as the number of subjects surviving to older ages increases in future generations with longer life expectancy. For example, if life expectancy of men increased to match the current life expectancy of women, so too would the male incidence rate of Alzheimer's disease.

Most importantly, the model allows us to predict the impact of preventive neuroprotective strategies on incidence rates of Alzheimer's disease [61]. Cell-rescuing neuroprotective therapies are expected to slow down the rate of neuron loss. As the probability that a neuron can be rescued from Alzheimer-related pathology is not diminished by age [58], the model can be easily accommodated to estimate the effect of cell-rescuing neuroprotection on incidence rates of Alzheimer's disease. It was found that even relatively modest neuroprotective effects on BCU counts can lead to dramatic reductions in incidence rates of Alzheimer's disease. Nonetheless, these results could only be obtained if such ‘hypothetical’ neuroprotective treatment were universally applied to the general population (with the additional problem of treatment-related side effects).

A more realistic neuroprotective strategy, which has important implications for public health decisions on putative preventive methods for Alzheimer's disease, refers to measures aimed at increasing the cognitive reserve. There is a growing body of evidence that higher levels of education and intellectual skills are associated with less cognitive impairment [9]–[14]. Intellectual activity likely improves the efficiency of brain networks and may also increase the level of redundancy in BCU circuits (i.e., more BCU need to be lost to reach the same level of cognitive deterioration) [10]. The model shows that a 5% increase in the cognitive reserve would prevent one third of Alzheimer's cases in the world. This result suggests that health policies aimed at increasing the level of education in the general population, as well as programs implementing cognitive training interventions, are likely the most effective method of preventing Alzheimer's disease. In fact, preliminary results from randomized controlled trials have already shown that cognitive stimulation slows down cognitive decline [62], [63].
